# Pharmacokinetics, Tissue Distribution, and Anti-Lipogenic/Adipogenic Effects of Allyl-Isothiocyanate Metabolites

**DOI:** 10.1371/journal.pone.0132151

**Published:** 2015-08-28

**Authors:** Yang-Ji Kim, Da-Hye Lee, Jiyun Ahn, Woo-Jae Chung, Young Jin Jang, Ki-Seung Seong, Jae-Hak Moon, Tae Youl Ha, Chang Hwa Jung

**Affiliations:** 1 Department of Food Biotechnology, Korea University of Science and Technology, Seongnam 463–746, Republic of Korea; 2 Metabolic Mechanism Research Group, Korea Food Research Institute, Seongnam 463–746, Republic of Korea; 3 Department of Genetic Engineering, Sungkyunkwan University, Suwon 440–746, Republic of Korea; 4 Department of Food Science & Technology, Chonnam National University, Gwangju 500–757, Republic of Korea; Bambino Gesù Children Hospital, ITALY

## Abstract

Allyl-isothiocyanate (AITC) is an organosulfur phytochemical found in abundance in common cruciferous vegetables such as mustard, wasabi, and cabbage. Although AITC is metabolized primarily through the mercapturic acid pathway, its exact pharmacokinetics remains undefined and the biological function of AITC metabolites is still largely unknown. In this study, we evaluated the inhibitory effects of AITC metabolites on lipid accumulation *in vitro* and elucidated the pharmacokinetics and tissue distribution of AITC metabolites in rats. We found that AITC metabolites generally conjugate with glutathione (GSH) or *N*-acetylcysteine (NAC) and are distributed in most organs and tissues. Pharmacokinetic analysis showed a rapid uptake and complete metabolism of AITC following oral administration to rats. Although AITC has been reported to exhibit anti-tumor activity in bladder cancer, the potential bioactivity of its metabolites has not been explored. We found that GSH-AITC and NAC-AITC effectively inhibit adipogenic differentiation of 3T3-L1 preadipocytes and suppress expression of PPAR-γ, C/EBPα, and FAS, which are up-regulated during adipogenesis. GSH-AITC and NAC-AITC also suppressed oleic acid-induced lipid accumulation and lipogenesis in hepatocytes. Our findings suggest that AITC is almost completely metabolized in the liver and rapidly excreted in urine through the mercapturic acid pathway following administration in rats. AITC metabolites may exert anti-obesity effects through suppression of adipogenesis or lipogenesis.

## Introduction

The glucosinolates are organosulfur compounds that are present in cruciferous vegetables such as cabbage, kale, and broccoli, with particularly high concentrations found in mustard, horseradish, and wasabi [1]. When a plant is mechanically damaged, isothiocyanates are generated from glucosinolates as products of hydrolyzation by plant myrosinase [2]. Recent studies show that glucosinolates are converted to different hydrolysis product by human gut bacteria and differently metabolized depending on individual gut bacteria composition [3,4]. Isothiocyanates such as sulforaphane (SFN), allyl-isothiocyanate (AITC), phenethyl-isothiocyanate (PEITC), and benzyl-isothiocyanate (BITC) are recognized as phytochemical chemopreventive agents that have been shown to inhibit proliferation and to induce apoptosis through G2/M arrest in bladder cancer UM-UC-3 cells [1,5–8]. AITC also induced apoptosis through down-regulation of anti-apoptotic Bcl-2 and Bcl-xl in PC3 and LNCaP cells [9]. Isothiocyanates have impressive chemopreventive properties in animal models [10]. Recent epigenetic studies report that isothiocyanates inhibit histone acetylation in human colon, prostate, and breast cancer cells [11,12]. In general, epigenetic modifications such as DNA methylation and histone acetylation have been associated with various types of cancers. Epidemiological research also suggests that high consumption of cruciferous vegetable is associated with a decreased risk of cancer [13]. These results suggest that isothiocyanates in cruciferous vegetables could be used to prevent human cancers. Additionally, isothiocyanates suppress nitric oxide (NO) production and NO synthase (iNOS) expression through attenuation of NF-κB activation in lipopolysaccharide-treated cells [14–16]. Taken together, these findings suggest that isothiocyanates may act as anti-inflammatory and anti-cancer agents.

Various drug transporters, such as the ATP-binding cassette (ABC) transporters, play significant roles in the absorption and disposition of most drugs. For many years, cancer researchers have focused on members of the ABC superfamily that play crucial roles in the development of resistance by mediating efflux of anticancer agents. Recent studies have indicated that phytochemicals can serve as substrates and modulators of proteins involving in efflux of anticancer agents. For example, isothiocyanates are rapidly exported as GSH- and cysteinylglycine-congugated forms by multidrug-resistance-associated protein 1 (MRP1) and P-glycoprotein (P-gp). These findings suggest that isothiocyante such as AITC could influence the pharmacokinetics of drug used in anti-cancer therapy by interacting with ABC transporters.

AITC is known to be responsible for the characteristic pungent aroma of some cruciferous vegetables, including radish and horseradish. AITC is produced by the hydrolysis of glucosinolate precursor sinigirin and account for the pungent taste of wasabi (193.78 mg/100 g of fresh rhizome) and horseradish (165.81 mg/100 g fresh root), in which it is present at high concentrations [17]. Due to the potentially beneficial impact of AITC on human health, it has become a focus of interest in the medical reteach community. However, the beneficial effects of AITC are dependent on its uptake and disposition in tissues. Previously published work has shown AITC to be rapidly cleared within 24 h of oral administration of ^14^C-labeled AITC in rats and mice, with relatively high deposition in bladder tissue compared to that in other organs [18]. AITC absorbed *in vivo* is metabolized through the mercapturic acid pathway in the liver, with the initial conjugation of the–N = C = S group with the cysteine thiol of glutathione resulting in the formation of a corresponding glutathione conjugate (GSH-AITC), which is further metabolized to an *N*-acetyl-*S*-(*N*-allylthiocarbamoyl)cysteine (NAC-AITC) conjugate [19,20]. While the formation of AITC metabolites is recognized to proceed through the mercapturic acid pathway after absorption, the pharmacokinetics of the metabolites has not elucidated thus far.

Sulforaphane inhibits adipocyte differentiation of 3T3-L1 preadipocytes by blocking clonal expansion via cell arrest, stimulating lipolysis via hormone-sensitive lipase activation and regulating AMPK signaling [21–23]. A previous study demonstrated that wasabi (*Wasabia japonica* Matsum.), which contain several pungent constituents, including AITC, suppressed obesity in mice on a high-fat diet, possibly due to suppression of lipid accumulation in the liver and white adipose tissue [24]. Several studies have shown that AITC increases triglyceride hydrolysis in the serum and, enhances lipolysis in adipocytes, and improves glucose intolerance in intraperitoneal glucose tolerance test (IPGTT) [25,26]. AITC is an attractive candidate agent for cancer chemoprevention. However, little is known regarding the role of isothiocyanates in lipid metabolism. We have recently shown that AITC reduces body weight and improves insulin sensitivity by correcting high-fat diet-induced mitochondrial dysfunction [27]. Despite the recent interest in the pharmacological activity of AITC, very few studies have focused on the potential bioactive properties of AITC metabolites. Following, the absorption of an orally administered dose, AITC is metabolized and the resulting metabolites are distributed throughout the body. A study evaluating the activity of AITC metabolites and their molecular mechanisms is warranted.

In this study, we first identified AITC metabolites using liquid chromatography-tandem mass spectroscopy (LC-MS/MS) in plasma following oral administration of AITC. Subsequently, we synthesized AITC metabolites to study their pharmacokinetics and biological activity. We evaluated the anti-lipogenic and anti-adipogenic effects of AITC metabolites *in vitro* and investigated the pharmacokinetics and tissue distribution of AITC metabolites *in vivo* using high-performance liquid chromatography (HPLC) analysis.

## Materials and Methods

### Chemicals and reagents

AITC (A33205), phosphoric acid, oleic acid (O03008), isobutylmethylxanthine (IBMX, I7018), dexamethasone (D4902), insulin (#16634), and Oil Red O (O0625) were obtained from Sigma-Aldrich (St Louis, MO, USA). HPLC-grade water, acetonitrile, and methanol were purchased from Fisher Scientific (Pittsburgh, PA, USA). All other reagents were of analytical grade. Antibodies against PPAR-γ (sc-7273), SREBP-1c (sc-366) and secondary antibodies were purchased from Santa Cruz Biotechnology (Santa Cruz, CA, USA). Antibodies against FAS (#3180), β-actin (#4967), C/EBPα (#2295s), p-S6K1 (#9205), and S6K1 (#9202) were purchased from Cell Signaling Technology (Danvers, MA, USA). Dulbecco’s modified Eagle’s medium (DMEM), fetal bovine serum (FBS), bovine calf serum (CS), sodium pyruvate, and penicillin-streptomycin were obtained from Gibco BRL (Grand Island, NY, USA).

### Treatment of animals

Male Sprague-Dawley rats with an average weight of approximately 250 g were purchased from Orient Bio Inc. (Seoul, South Korea) and acclimatized to the laboratory conditions (12:12 h light-dark cycle, temperature of 24°C, and humidity of 55%) for 1 week. Animals were provided the American Institute of Nutrition-93 (AIN-93) diet and water ad libitum during acclimation. On the dosing day, rats were fasted for 6 h and then administered AITC (25 mg/kg of body weight) as a suspension (1 mL) in polyethylene glycol using an oral zonde needle. After a single-dose oral administration of AITC, rats were sacrificed under anesthesia (80 mg/kg ketamine + 8 mg/kg xylazine) at different time points after dosing. Blood samples were collected from the abdominal aorta in heparinized syringes. Samples were immediately centrifuged at 3,000 × g for 10 min and the resulting plasma samples were kept frozen at -80°C until analysis. The liver, heart, spleen, kidney, and lung were rapidly collected from each animal at different time points, individually wrapped in aluminum foil, snap-frozen in liquid nitrogen, and stored frozen at -80°C until use. Animal studies were conducted in accordance with institutional and national guidelines, and all experimental procedures were approved by the Korea Food Research Institute Animal Care and Use Committee (KFRI-IACUC, #2013–0018).

### AITC extraction from plasma

Methanol (700 μL) was added to 300 μL of serum and the mixture was mixed by vortexing for 5 min. After centrifugation at 15,700 × *g* for 15 min at 4°C, the supernatant was collected and injected onto the HPLC or LC-MS system. The internal standards (synthesized metabolites) were added to the plasma samples for validation.

### Tissue extraction

Dissected tissue was thawed, rinsed with ice-cold saline, blotted dry, and weighed. Each tissue sample (100 mg) was homogenized in 100 μL phosphate-buffered saline (PBS) using homogenizer (MP Fastprep instrument, MP Biomedical, Solon, OH, USA). The homogenates were transferred into clean tubes and mixed with 700 μL of methanol by vortexing for 5 min. Homogenates were centrifuged at 15,700 × *g* for 15 min at 4°C, and the supernatants were collected and evaporated to dryness at room temperature under a stream of nitrogen gas. The residue was diluted with 100 μL 70% methanol.

### LC-LTQ Orbitrap analysis

Chromatographic separation was performed using the Accela ultra performance liquid chromatography (UPLC) system (Thermo Scientific, San Jose, CA) with an ACQUITY BEH C_18_ column (Waters Corporation, Milford, MA, USA; 1.7 μm, 150 × 2.1 mm). The mobile phase was 0.1% formic acid in water (A) and acetonitrile. The gradient elution was linearly programmed as follows: 0 min 10% B, 10 min 90% B, 10.1 min 100% B, 12 min 0% B, with the mobile phase flow rate set at 0.4 mL/min. Mass spectroscopy (MS) analysis was carried out using an LTQ Orbitrap XL instrument (Thermo Scientific, San Jose, CA). The ionization and capillary voltages were set to 4.2 Kv and 35V, respectively. The capillary temperature was set at 300°C. MS data were collected in the *m/z* 100–1300 scan range in positive ion mode. Fragment ions for tandem mass spectroscopy (MS/MS) acquisition were produced by collision-induced-dissociation (CID) at of 45 eV and data acquisition was performed using the Xcalibur software (Thermo Finnigan).

### HPLC analysis

HPLC analysis was performed using a Jasco HPLC system equipped with a Jasco UV-2089 plus quaternary gradient pump, a Jasco MD-2010 plus multiple-wave-length detector, and Borwin chromatography software, version 1.5 (Jasco Inc., Easton, MD, USA). Separation was achieved using an XTerra RP18 column (4.6 × 250mm, 5 μM, Waters, Milford, MA, USA). Elution was performed at a flow rate of 1.0 mL/ min at 30°C using 1% (v/v) phosphoric acid (solvent A) and acetonitrile (solvent B) as mobile phase. The solvent gradient was changed as follows: from 100% A to 95% A over 10 min, to 85% A over 10 min, to 70% A over 10 min, to 65% A over 5 min, to 90% A over 5 min, and to 100% A over 5 min, followed by 5 min of maintenance at 100% A.

### Synthesis of GSH-AITC (S-(N-allylthiocarbamoyl)-L-glutathione)

GSH (6 mg) was dissolved in 0.9mL of 1N HCl and 1N NaOH was added to adjust the pH to 6.5. Methanol (1 mL) was added to the solution. AITC (21 μL) was added to the GSH solution with stirring. The reaction mixture was stirred at room temperature for 3 days. The volume of the reaction mixture was reduced by half under vacuum and 1ml of tetrahydrofuran (THF) was added. The precipitate was collected by centrifugation (4000 × *g*, 1 min) and dried under vacuum. The product was isolated by flash column chromatography using a mixture of CHCl_3_ and methanol (1:1) as an eluent. The solvent was removed under vacuum using a rotary evaporator.

NMR Assignments: ^1^H-NMR (500 MHz, D_2_O+DMSO-d6) δ 2.075 (2H, dd, Glu-CH-CH_2_-CH_2_), 2.431 (2H, m, Glu-CH-CH_2-_CH_2_), 3.497 (1H, dd, Cys-CH-CH_2_), 3.703 (1H, t, Glu-CH), 3.844 (1H, dd, Cys-CH-CH_2_), 3.878 (2H, s, Gly-CH_2_), 4.262 (2H, d, allyl CH_2_), 4.724 (1H, dd, Cys-CH), 5.157–5.191 (2H, vinyl CH = CH_2_), 5.846 (1H, dq, vinyl CH = CH_2_). ESI-MS (positive mode): [M+H]^+^ = 407.7.

### Synthesis of NAC-AITC (N-acetyl-S-(N-allylthiocarbamoyl)- l-cysteine)

NAC (44 mg) was dissolved in 150 μL of 1N NaOH and added to 0.8 mL of water. The pH was adjusted to 8.0 by adding 1N NaOH. AITC (50 μL in 1 of mL THF) was added to the NAC solution with stirring. The reaction mixture was stirred at room temperature overnight. Water (5 mL) was added to the reaction mixture and the pH was adjusted to 3 by adding 0.5N HCl. The crude product was extracted with ethyl acetate 3 times. The collected ethyl acetate solution was dried with MgCl_2_ for 3 h. The solvent of the filtrate was removed under vacuum. The product was isolated by flash column chromatography using a mixture of CHCl_3_ and methanol (5:1) as an eluent. The solvent was removed under vacuum using a rotary evaporator.

NMR Assignments: ^1^H-NMR (500 MHz, DMSO-d6) δ 1.833 (3H, S, CH_3_-CO), 3.325 (1H, dd, J = 13.5, 9 Hz, Cys-CH-CH_2_), 3.767 (1H, dd, J = 13.5, 5 Hz, Cys-CH-CH_2_), 4.220 (2H, m, allyl CH_2_), 4.405(1H, ddd, J = 9, 8.5, 5 Hz, Cys-CH), 5.133 (1H, dq, J = 10, 1.5 Hz, cis-vinyl-CH_2_), 5.184 (1H, dq, J = 17.5, 1.5 Hz, trans-vinyl-CH_2_), 5.845 (1H, m, CH_2_ = CH), 8.282 (1H, d, J = 8.5 Hz, CH_3_CONH). ESI-MS (negative mode): [M—H]^-^ = 261.4.

### Analysis of pharmacokinetics

Non-compartmental (mode-independent) nutrikinetic parameters were derived using PK Solutions software version 2.0 (Summit Research Services, Montrose, CO, USA). The maximum plasma concentration (C_max_), time to maximum plasma concentration (T_max_), and the elimination half-life (t_1/2_) were estimated from the plasma concentration-time curve. The area under the curve (AUC) for the plasma concentration over time was calculated using the trapezoidal rule.

### Adipocyte differentiation

3T3-L1 pre-adipocyte cells were purchased from the ATCC (Manassas, VA, USA). Cells were maintained in DMEM supplemented with 10% calf serum (CS), 100 U/mL penicillin and 100 μg/mL streptomycin at 37°C in a 5% CO_2_ incubator. The cells were seeded onto a 6-well plate at a density of 4 × 10^5^ cells per well, and cell differentiation was induced. In brief, beginning at 2 days post-confluence (day 0), cells were incubated for 2 days in DMEM containing 0.5 mM 3-isobutyl-1-methylxanthine (IBMX), 1 μM dexamethasone, 1 μg/mL of insulin, and 10% fetal bovine serum (FBS). After induction, the medium was replaced with DMEM containing 10% FBS and 1 μg/mL of insulin, and the cells were incubated for 2 more days, after which the cells were maintained in DMEM containing 10% FBS until they reached maturity. From days 0 to 2, the cells were exposed to the AITC metabolites.

### TG accumulation in hepatocytes

HepG2 (human hepatoma cell line) and Hepa 1–6 (mouse hepatoma cell line) cells were obtained from ATCC (Manassas, VA, USA) and grown in high glucose DMEM supplemented with 10% FBS, 100 U/mL penicillin, and 100 μg/mL streptomycin. For examination of lipid accumulation in hepatocytes, the cells were treated with AITC metabolites in presence of 100 μM oleic acid for 24h.

### Oil Red O staining

Oil Red O staining was performed on day 8. Prior to quantification, the cells were fixed in 10% neutral formalin for 1 h at room temperature, washed with PBS, and stained for 1 h with 0.5% Oil Red O in 60% isopropanol. After staining, the cells were washed with distilled water and observed under a fluorescence microscope. The Oil Red O stain was extracted from the cells with 1 mL of 100% isopropanol for 10 min. The optical density was measured at a wavelength of 490 nm using microplate reader (Tecan Infinite 200 PRO, Männedorf, Switzerland).

### Western blotting

3T3-L1 adipocytes were scraped into a lysis buffer containing 40 mM HEPES (pH 7.4), 120 mM NaCl, 1 mM EDTA, 50 mM NaF, 1.5 mM Na_3_VO_4_, 10 mM β–glycerophosphate, and 1% Triton X-100, supplemented with EDTA-free phosphatase and a protease inhibitor cocktail (#78441; Thermo Scientific, Waltham, MA, USA). Next, the cells were lysed by sonication twice for 15 sec on ice using a sonicator (Misonix, Farmingdale, NY, USA). After centrifugation at 10,000 × *g* for 20 min at 4°C, the supernatants were boiled in sodium dodecyl sulfate (SDS) loading buffer at 90°C for 5 min, after which they were loaded (20 μg protein per lane) onto 8% or 12% Tris-glycine gels. Following electrophoresis, the proteins were transferred onto polyvinylidene fluoride (PVDF) membranes, which were blocked using 5% skim milk in TBST buffer (20 mM Tris, pH 8.0, 125 m NaCl, 0.5% Tween 20) for 1 h. The blocked membranes were incubated with primary antibodies in TBST overnight at 4°C, followed by incubation with secondary antibodies for 1 h at room temperature. Finally, the membranes were visualized using a chemiluminescence reagent (Amersham Bioscience, Piscataway, NJ, USA).

### Statistical analysis

Differences between groups were evaluated using one-way analysis of variance (ANOVA) with Prism5 software (version 5, GraphPad software, San Diego, CA, USA). The Bonferroni post-hoc correction for multiple comparisons was used when significant differences were identified using ANOVA (*p* < 0.05). The data are expressed as mean ± standard deviation (SD).

## Results

### NAC-AITC and GSH-AITC are the primary AITC metabolites in rat plasma after oral administration

**Fig 1:**
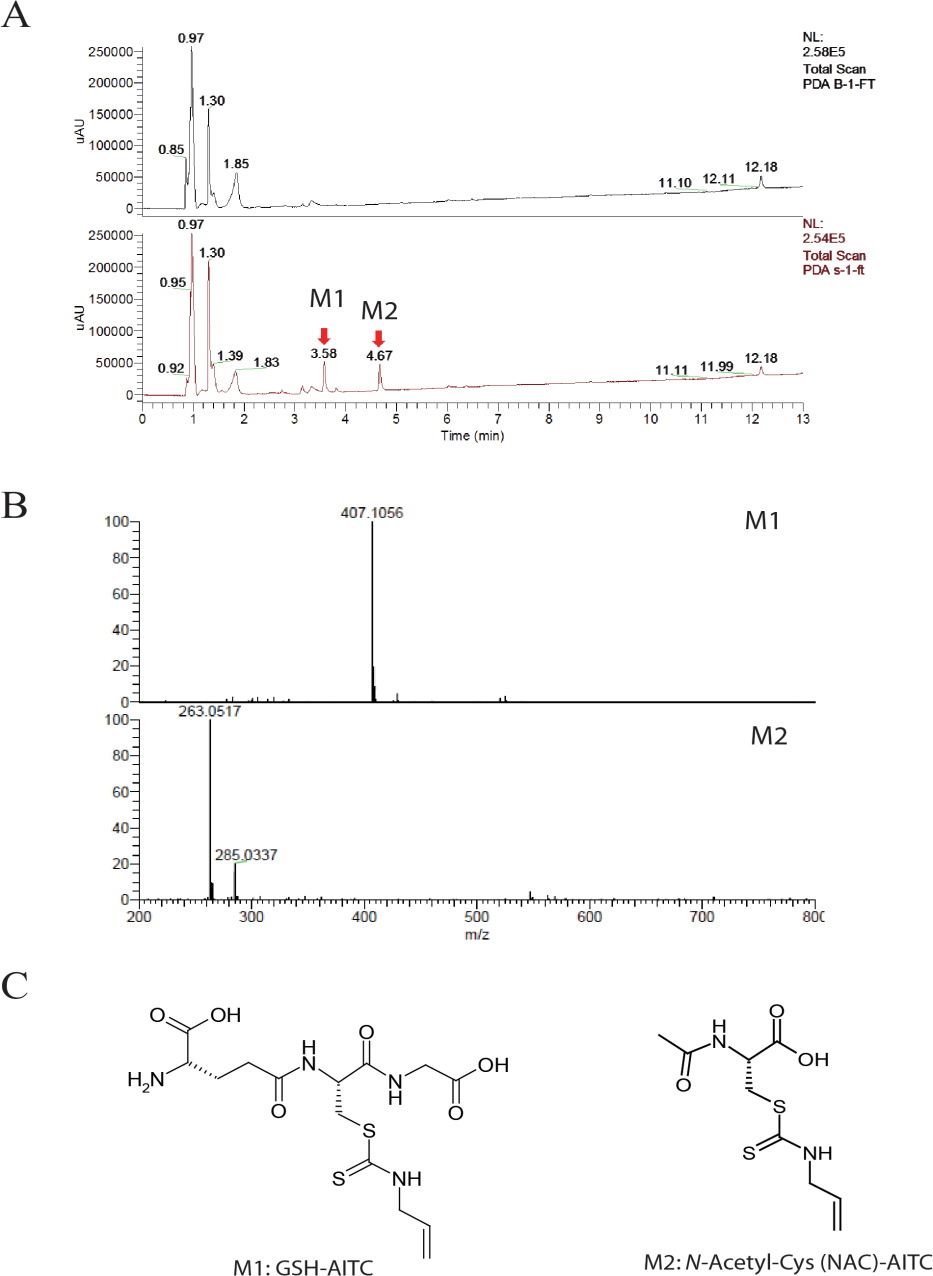
Analysis of AITC metabolites in rat plasma following oral administration of AITC using liquid chromatography-tandem mass spectroscopy (LC-MS/MS). (A) Spectra of blank and AITC-supplemented samples. (B) LC-MS/MS fragmentation. The lines indicate the predicted metabolites composition. (C) Structures of the identified metabolites.

Identification of AITC metabolites was accomplished by LC-LTQ Orbitrap analysis of plasma samples collected following oral administration of AITC. We compared blank and post-administration samples and found 2 candidate compounds that eluted at retention times of 3.58 and 4.67 min, which were considered to represent AITC and its metabolites (**Fig 1A**). The measured molecular weights of the 2 metabolites were *m/z* 407.1056 [M+H]^+^ and *m/z* 263.0517 [M+H]^+^ (**Fig 1B**). Based on the MS/MS spectra obtained, the proposed chemical compositions of compounds M1 and M2 were C_14_H_23_O_6_N_4_S_2_ and C_9_H_15_O_3_N_2_S_2_, respectively, reflecting metabolism by the mercapturic acid pathway. M1 and M2 were proposed to be GSH-AITC and NAC-AITC. The proposed structures of the 2 metabolites are shown in **Fig 1C**. These results suggest that AITC is metabolized quickly and almost completely after oral administration.

To study the pharmacokinetics and biological activities of the identified AITC metabolites, we synthesized them according to the procedures described in the Materials and Methods section. LC-MS data showed that the molecular weights of the synthesized metabolites were consistent with M1 and M2 (data not shown). Previously reported NMR spectrum data also supported the identification of one of the synthesized compounds as M2, the NAC conjugate of AITC [28]. Although we were unable to find corresponding NMR spectrum data for GSH-AITC, the other synthesized compound was identified as M1, the GSH conjugate of AITC, based on its UV absorption pattern and retention time (data not shown).

### AITC is quickly absorbed, distributed, and excreted

**Fig 2:**
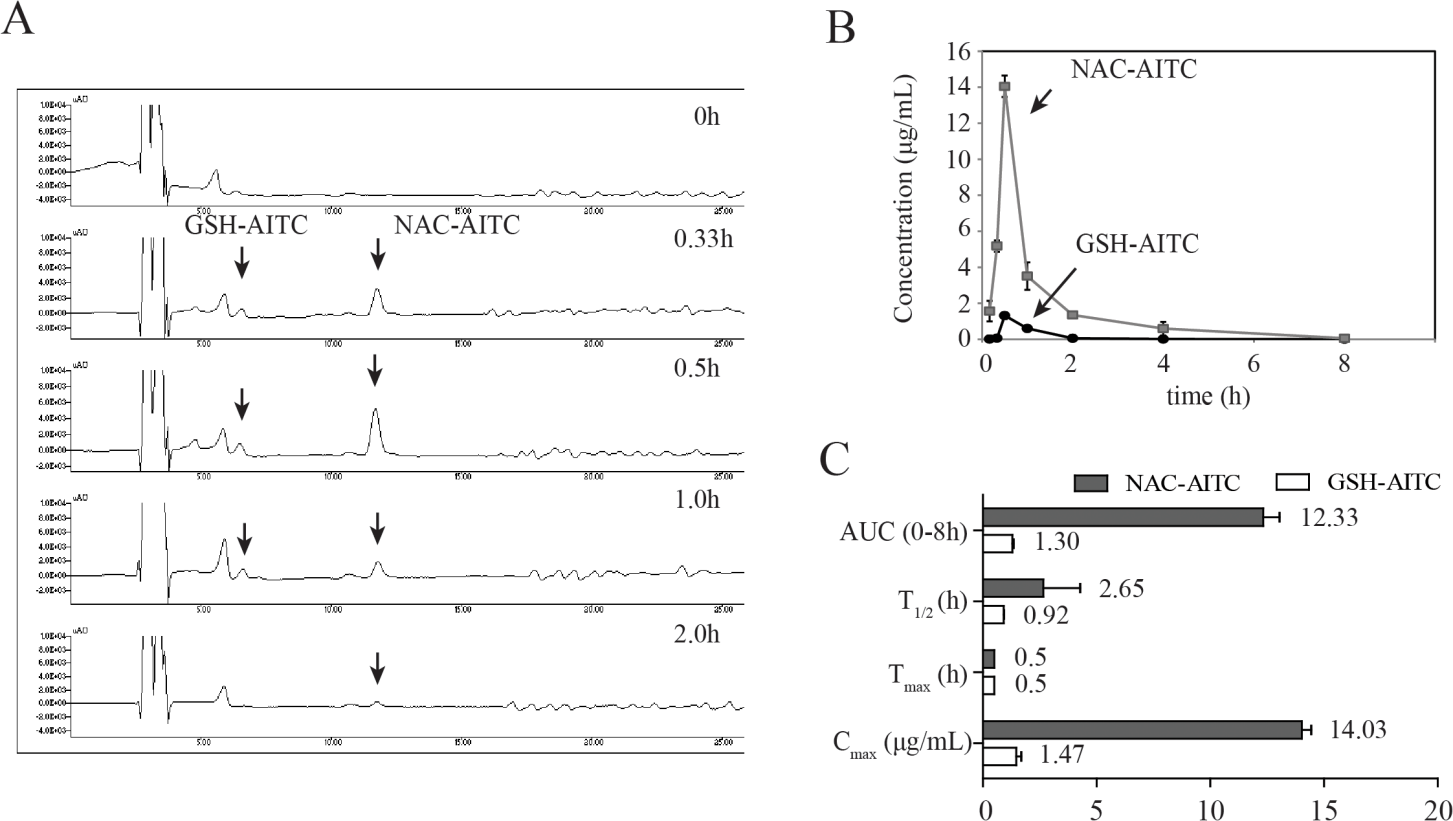
Pharmacokinetic analysis of GSH-AITC and NAC-AITC in rat plasma. (A) HPLC chromatograms of AITC metabolites in plasma obtained at different time points following oral administration of AITC obtained by measuring absorbance at a wavelength of 250 nm. (B) Plasma concentration-time profiles of AITC metabolites. (C) Pharmacokinetic parameters of AITC metabolites in rat plasma following oral administration. C_max_: maximum recorded concentration; T_max_: time taken to reach C_max_; AUC (area under the curve): a measure of the exposure to the drug; T_1/2_: elimination half-life. All values are reported as mean ± SD (n = 5).

The concentration of AITC metabolites in the plasma were measured by HPLC following oral administration of AITC at different time points up to 8 h post-administration. GSH-AITC and NAC-AITC were found to elute without any interference from endogenous components in the plasma (**Fig 2A**). The correlation coefficient of the calibration curve was above 0.99, suggesting good linearity within the GSH-AITC concentration range of 0.1–4 μg/mL and the NAC-AITC concentration range of 2.5–60 μg/mL. The inter- and intra-day precision and accuracy were determined by replicate analysis of the samples at 3 concentrations of each metabolite: 1, 2, and 4 μg/mL GSH-AITC and 20, 40, and 60 μg/mL of NAC-AITC. The stability of the metabolites was determined at room temperature over 24 h. The precision and accuracy were found to be well within the acceptance range of 10% and the metabolites in the plasma were found to be stable for 24 h at room temperature (data not shown).

A validated analytical method was employed to study the pharmacokinetic profiles of AITC metabolites after oral administration of 25 mg/kg AITC dose to rats. The mean plasma concentration-time curves of the AITC metabolites and the corresponding pharmacokinetic parameters are shown in **Fig 2B** and **2C**. The pharmacokinetic analysis was performed with PK solution 2.0 software, using a non-compartmental model for data analysis. After oral administration of AITC, GSH-AITC and NAC-AITC were detected in the plasma. The T_max_ values for GSH-AITC and NAC-AITC were similar at 0.5h. The C_max_ values of GSH-AITC and NAC-AITC were 1.47 and 14.03 μg/mL, respectively. The areas under the curve of the concentration-time profile (AUC_0-8h_) of GSH-AITC and NAC-AITC were 1.3 and 12.33 μg·h/mL, respectively. These results indicate that NAC-AITC and GSH-AITC were rapidly formed and widely circulated, with NAC-AITC being the main metabolite observed following oral administration of AITC.

**Fig 3:**
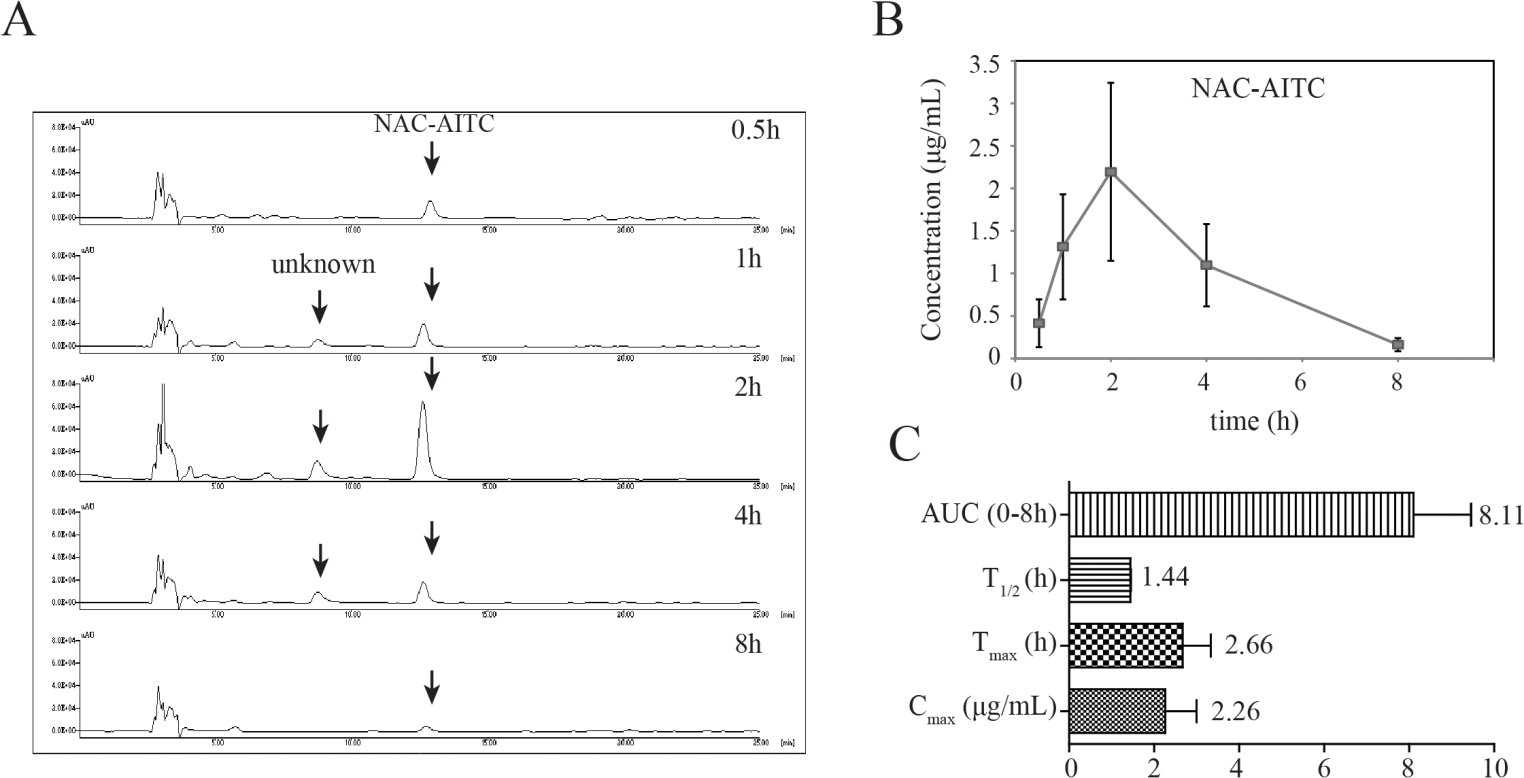
Pharmacokinetic analysis of GSH-AITC and NAC-AITC in rat urine. (A) HPLC chromatograms of AITC metabolites in urine obtained at different time points following oral administration of AITC. (B) Urine concentration-time profiles of NAC-AITC. (C) Pharmacokinetic parameters of NAC-AITC in urine. All values are reported as mean ± SD (n = 5).

In addition to our evaluations of plasma levels, we also determined the concentration of AITC metabolites in urine using HPLC. While NAC-AITC was successfully quantified, GSH-AITC was not detected in the urine samples (**Fig 3A**), possibly reflecting a low concentration of this metabolite in the urine. The mean urine concentration-time profiles of NAC-AITC are shown in **Fig 3B**. The NAC-AITC concentration reached a maximum level between 1 and 4 h following administration of AITC, with a C_max_ of 2.26 μg/mL. NAC-AITC was mostly excreted within 8 h (**Fig 3C**).

**Fig 4:**
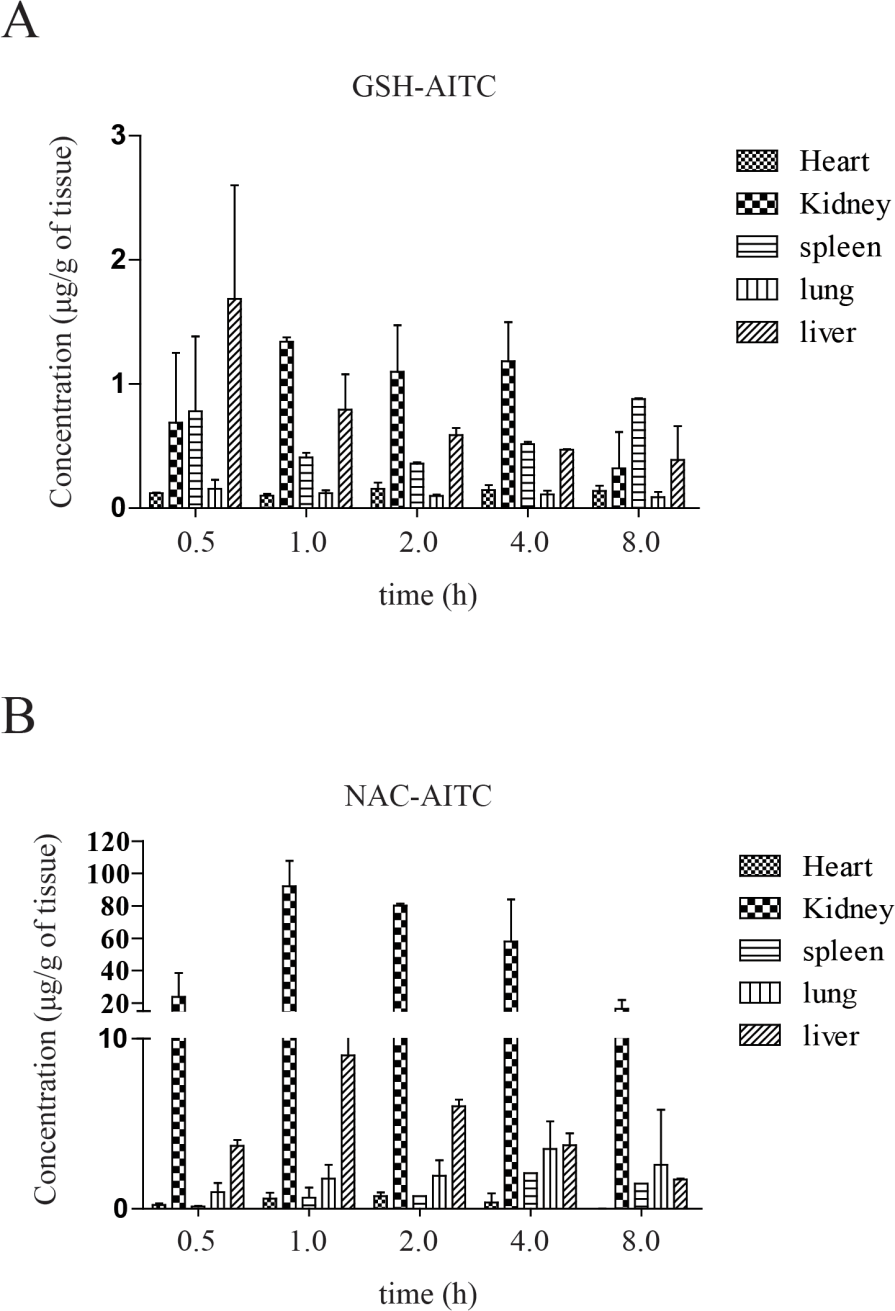
Tissue distribution of AITC metabolites after oral administration. Concentration of GSH-AITC (A) and NAC-AITC (B) were measured in several tissue types following AITC administration. AITC metabolites were extracted from the tissues at each time point (0.5, 1, 2, 3, and 8 h) and evaluated by HPLC.

To study the tissue distribution of AITC metabolites, rats were sacrificed and their organs were collected at different time points following oral administration of AITC. The assay demonstrated that the metabolites underwent rapid and wide distribution to tissues within the studied time period (**Fig 4A** and **4B**). The greatest GSH-AITC deposition by tissue weight was observed in the liver, followed by the kidney, spleen, heart, and lung, whereas the greatest NAC-AITC deposition (by tissues weight) was observed in the kidney. These findings indicate that the metabolites distributed mainly into tissues with abundant blood supply, implying that the blood flow (perfusion rate) of an organ is the key factor affecting the distribution of AITC metabolites. Additionally, our observations suggest that AITC metabolites distributed in various tissues could have diverse effects on the body.

### AITC metabolites suppress adipocyte differentiation of 3T3-L1 cells

**Fig 5:**
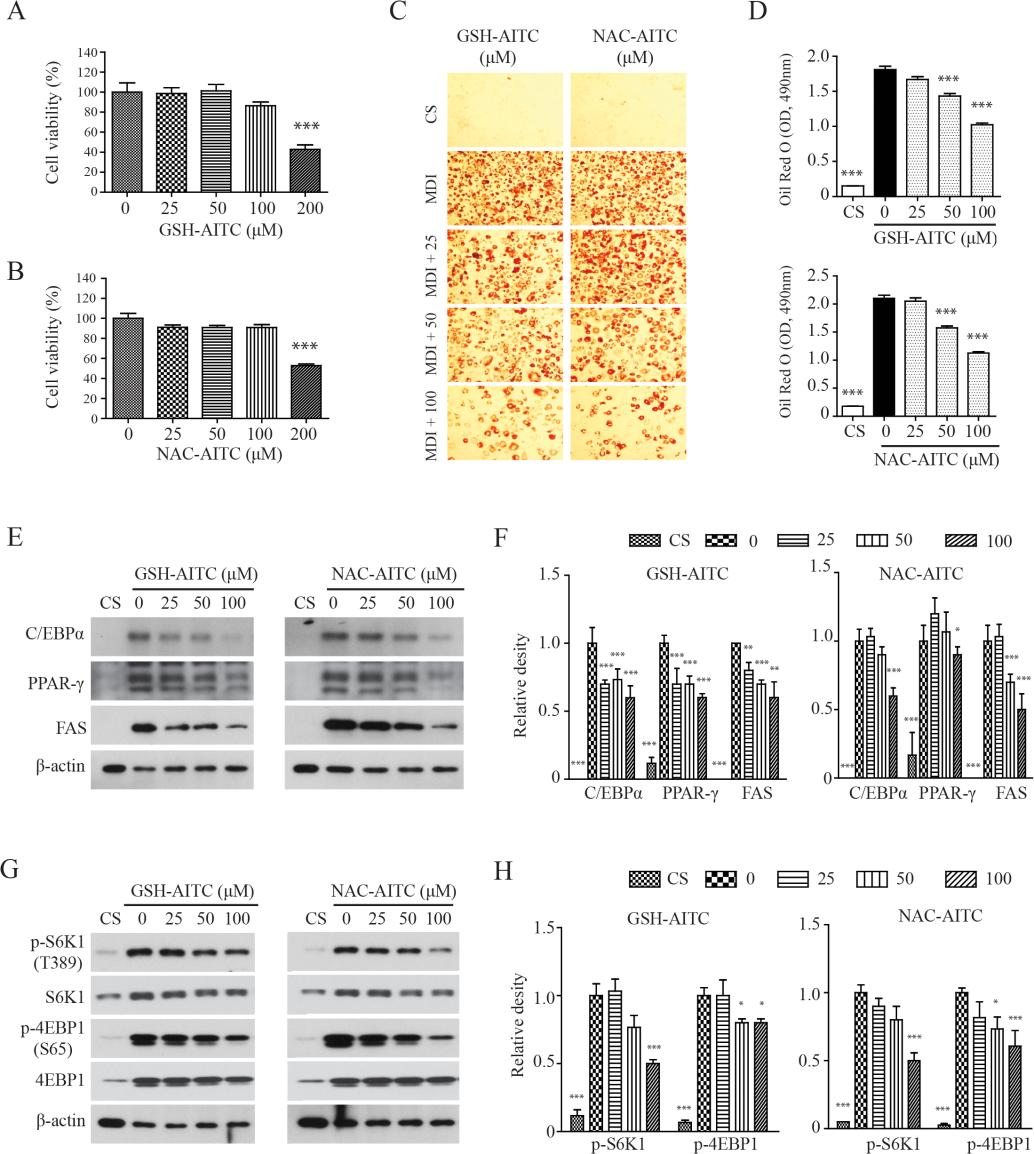
Anti-adipogenic effects of GSH-AITC and NAC-AITC in 3T3-L1 cells. Cell viability following treatment with GSH-AITC (A) and NAC-AITC (B) was assessed from day 0 to 2 (48h). The cell viability was determined using the cell counting kit-8 (CCK-8). Data are expressed as percentages of the control values and shown as mean ± SD (n = 3). (C) GSH-AITC and NAC-AITC inhibited differentiation of preadipocytes. 3T3-L1 cells were treated with different concentration of AITC metabolites and stained with Oil Red O after 8 days of cell differentiation. (D) Quantitative analysis of the relative intensity of the Oil Red O stain. The intracellular triglyceride levels were indexed by quantifying the Oil Red O extracted from the cells by spectroscopy performed at an excitation wavelength of 490 nm. Values are reported as means ± SD (n = 3, *** *p* < 0.001 compared to vehicle). (E) GSH-AITC and NAC-AITC suppressed the expression of proteins associated with adipogenesis. Cell lysates were harvested into lysis buffer and analyzed by western blotting. To compare and quantify levels of proteins, the density of each band was measured using ImageJ software with β–actin as an internal reference. (F) GSH-AITC and NAC-AITC inhibited mTOR activity during cell differentiation.

Although it has been shown that AITC reduces high-fat diet-induced obesity [27], active metabolites of AITC were not identified in previous studies, and their potential to inhibit adipogenesis has not been explored. We performed *in vitro* analyses to determine whether GSH-AITC and NAC-AITC inhibit 3T3-L1 pre-adipocyte differentiation. The viability of 3T3-L1 cells was not affected by exposure to either AITC metabolite at a concentration of 100 μM (**Fig 5A** and **5B**). Both metabolites significantly suppressed MDI (3-IBMX, dexamethasone, and insulin)-induced differentiation of 3T3-L1 preadipocytes in a dose-dependent manner (**Fig 5C** and **5D**). Western blotting results indicated that GSH-AITC and NAC-AITC reduced levels of PPAR-γ, C/EBPα, and FAS, proteins known to be up-regulated during adipogenesis (**Fig 5E**). These results strongly suggest that GSH-AITC and NAC-AITC suppress the expression of genes related to adipogenesis.

### AITC metabolites inhibit adipogenesis through the inhibition of Akt-mTORC1

The mammalian target of rapamycin complex 1 (mTORC1) plays a critical role in adipogenesis, because insulin stimulates adipogenesis through the AKT-TSC2-mTORC1 pathway [29,30]. We therefore investigated the possibility that AITC metabolites affect mTORC1 activation. Treatment with either NAC-AITC or GSH-AITC reduced the levels of S6K1 phosphorylation on Thr389, the mTOR-dependent phosphorylation site, during 3T3-L1 adipocyte differentiation (**Fig 5F**). The AITC metabolites also inhibited phosphorylation of 4EBP1, a target of mTORC1 [31,32]. Therefore, AITC metabolites may inhibit adipogenesis by regulating mTORC1 activity.

### AITC metabolites reduce oleic acid-induced lipid accumulation

**Fig 6:**
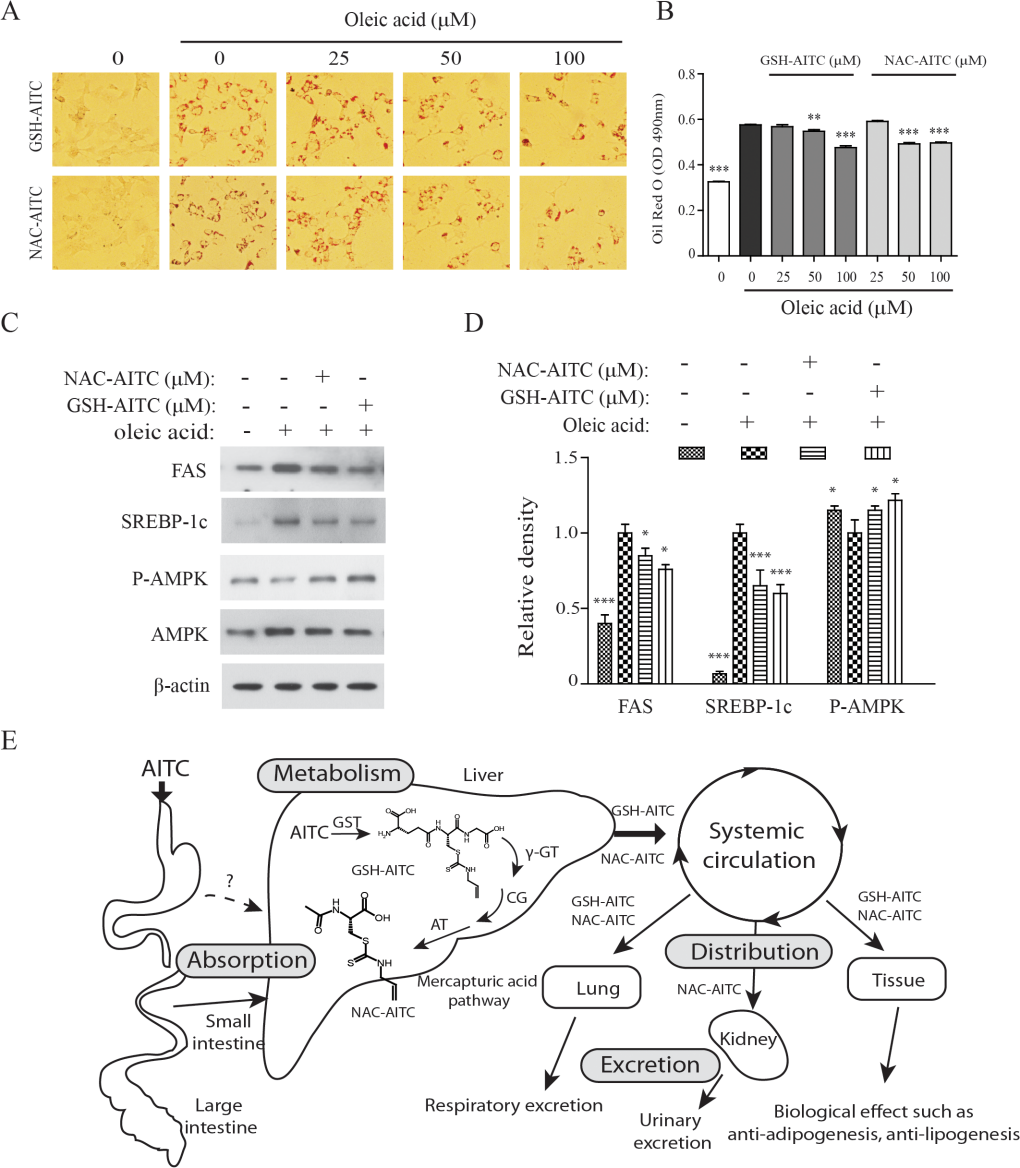
Inhibitory effects of GSH-AITC and NAC-AITC on oleic acid-induced lipid accumulation. Hepa 1–6 cells were treated with the indicated concentrations of GSH-AITC and NAC-AITC in the presence or absence of 100 μM oleic acid. Cells were stained with Oil Red O (A). The intracellular triglyceride levels were indexed by quantifying the Oil Red O extracted from the cells by spectroscopy performed at an excitation wavelength of 490 nm (B). The SCD1, FAS, and AMPK levels in cell lysates were detected by western blot analysis. To compare and quantify levels of proteins, the density of each band was measured using ImageJ software with β–actin as an internal reference (C). Values are reported as means ± SD (n = 3, ** *p* < 0.01; *** *p* < 0.001 compared to oleic acid only). (D) Proposed metabolic pathway of AITC after oral administration of AITC.

We determined the effects of the identified AITC metabolites on oleic acid (OA)-induced lipid accumulation in hepatocytes. In our previous study, we showed that AITC supplementation reduced hepatic steatosis in mice with high-fat diet-induced obesity [27]. Consistent with our hypothesis that AITC metabolites are responsible for decreased lipid accumulation in hepatocytes, we found that incubation with AITC metabolites significantly decreased OA-induced lipid accumulation in Hepa 1–6 cells (**Fig 6A and 6B**). In addition, both metabolites decreased expression of markers hepatic lipogenesis such as fatty acid synthase (FAS) and sterol regulatory element binding protein (SREBP)-1 (**Fig 6C**). AMPK is a central regulator of the lipid biosynthetic pathway [33]. The AITC metabolites increased adenosine 5′-monophosphate (AMP)-activated protein kinase (AMPK) activity in both tested cell lines. The viability of Hepa 1–6 cells in this assay was not affected by incubation with either AITC metabolite at a concentration of 100 μM (data not shown). This result suggests that GSH-AITC and NAC-AITC may play an active role in the prevention of the development of fatty liver.

## Discussion

Isothiocyanates, such as sulforaphane (SFN), allyl-isothiocyanate (AITC), phenethyl-isothiocyanate (PEITC), and benzyl-isothiocyanate (BITC) possess numerous health-related effects, and these compounds have been shown to inhibit cancer and inflammation and improve insulin sensitivity [19,27,34]. In this study, we investigated the nutrikinetics and tissue distribution of AITC metabolites formed following oral administration of AITC and evaluated the lipogenic and adipogenic effects of AITC metabolites. Isothiocyanates are absorbed across intestinal cell membranes by passive diffusion and are conjugated to GSH through a process catalyzed by glutathione *S*-transferases (GSTs) [35]. These GSH conjugates are subsequently metabolized by γ-glutamytranspeptidase (γ-GT), cystinylglycinase (CG), and *N*-acetyltransferase (AT) to form *N*-acetylcysteine conjugates [36,37]. Consistent with previous reports showing AITC to be metabolized through the mercapturic acid pathway in the liver, we confirmed that AITC is metabolized quickly and converted to GSH-AITC and NAC-AITC, which are rapidly eliminated. We expected to detect other intermediate metabolites, such as Gly-Cys-AITC or Cys-AITC, in the liver after oral administration of AITC; however, such intermediate metabolites were not detected in our study. There are several potential explanations for this result, such as changes in polarity, rapid metabolism, and low stability. NAC-AITC was only detected in the kidney, and thus it may account for the inhibitory effects of AITC accumulation on bladder cancer. AITC metabolites were primarily distributed to organs with an abundant blood-supply, including the liver, kidneys, and heart, indicating that metabolite distribution may depend on the blood flow and perfusion rate of each organ. Luang-In and colleagues reported the remarkable result that human gut bacteria may influence the metabolites that are generated by glucosinolate metabolism. This finding suggests that the profile of glucosinolate-derived metabolites in humans may be dependent on the strains of bacteria present in the gut [4].

Although the process of biotransformation in the liver generally decreases the biological activity of compounds, some metabolites retain or gain pharmacological effectiveness [38]. Hepatic phase I (cytochrome P450 (CYP)) and hepatic phase II (UDP glucuronosyl transferase (UDPGT) and glutathione S-transferase (GST)) enzymes play important roles in xenobiotic metabolism. Exogenous compounds are often subject to xenobiotic metabolism and generally have better safety profiles than the parent molecules. Our recent study demonstrates that AITC supplementation reduces high-fat diet-induced body weight gain and inhibits hepatic steatosis. We initially believed that AITC elicited the observed anti-obesity effect. Unexpectedly, AITC itself (the parent molecule) was not detected in the plasma and tissues, because it is completely metabolized following administration. An investigation of the anti-adipogenic effects of AITC metabolites has shown that both GSH-AITC and NAC-AITC attenuate lipid accumulation by regulating adipogenesis-related genes such as PPAR-γ, C/EBPα, and FAS in 3T3-L1 cells. Recent studies suggest that mTORC1 plays a critical role in adipogenesis [29,30]. AITC metabolites reduced S6K1 phosphorylation on Thr389 and 4EBP1 phosphorylation on Thr37/46, which are both mTORC1-dependent phosphorylation sites, suggesting that AITC metabolites inhibit lipid accumulation in 3T3-L1 adipocytes by attenuating mTORC1 activity. AITC metabolites also significantly decreased hepatocyte triglyceride content following exogenous lipid supplementation with oleic acid. Therefore, GSH-AITC and NAC-AITC may inhibit adipose and hepatic lipid accumulation in the body.

Although our results show that AITC metabolites exert anti-lipogenic and adipogenic effects *in vitro*, several issue are raised by our findings. According to the work of Bruggeman and colleagues, around 9–15% of the GSH conjugate of AITC was converted to free AITC in cellular medium [39]. Therefore, the observed efficacy of GSH-AITC in our study may have been partially due to the presence of free AITC. However, Zhang’s work suggests that free isothiocyanate released from conjugated compounds is taken up by cells as a GSH-conjugate, which accumulates very rapidly to high intracellular concentration [40]. Although NAC-AITC is the main metabolite of AITC, little is known about intracellular uptake of NAC-AITC, and further research is needed to address the mechanisms involved in this process. Nevertheless, results show that AITC metabolites may have direct anti-lipogenic and adipogenic effects *in vitro*.

On the basis of these findings, we conclude that GSH-AITC and NAC-AITC are active metabolites with effects related to anti-adipogenesis and anti-lipogenesis. The mercapturic acid pathway, a sequence of metabolic reactions catalyzed by phase II enzymes, may promote the excretion of AITC and may regulate its optimal functionality (**Fig 6D**). Although AITC itself is more active than its GSH- and NAC-conjugated forms, its metabolites exhibit pharmacological activity, such as anti-obesity effects. Both GSH-AITC and NAC-AITC markedly inhibited the accumulation of lipids in adipocytes and hepatocytes *in vitro*. This is the first study showing that AITC metabolites exhibit anti-obesity effects *in vitro*.
